# A Systematic Approach to Multiple Breath Nitrogen Washout Test Quality

**DOI:** 10.1371/journal.pone.0157523

**Published:** 2016-06-15

**Authors:** Renee Jensen, Sanja Stanojevic, Michelle Klingel, Maria Ester Pizarro, Graham L. Hall, Kathryn Ramsey, Rachel Foong, Clare Saunders, Paul D. Robinson, Hailey Webster, Kate Hardaker, Mica Kane, Felix Ratjen

**Affiliations:** 1 Division of Respiratory Medicine, Hospital for Sick Children, Toronto, Canada; 2 University of Toronto, Toronto, Canada; 3 Pontificia Universidad Católica de Chile, Santiago, Chile; 4 Telethon Kids Institute, Perth, Australia; 5 Centre for Child Health Research, University of Western Australia, Perth, Australia; 6 School of Physiotherapy and Exercise Science, Curtin University, Perth, Australia; 7 University of North Carolina at Chapel Hill, Chapel Hill, United States of America; 8 Royal Brompton & Harefield NHS Foundation Trust, London, United Kingdom; 9 Department of Gene Therapy, Imperial College London, London, United Kingdom; 10 Department of Respiratory Medicine, The Children’s Hospital at Westmead, Sydney, Australia; 11 Discipline of Paediatrics and Child Health, University of Sydney, Sydney, Australia; University Children's Hospital Bern, SWITZERLAND

## Abstract

**Background:**

Accurate estimates of multiple breath washout (MBW) outcomes require correct operation of the device, appropriate distraction of the subject to ensure they breathe in a manner representative of their relaxed tidal breathing pattern, and appropriate interpretation of the acquired data. Based on available recommendations for an acceptable MBW test, we aimed to develop a protocol to systematically evaluate MBW measurements based on these criteria.

**Methods:**

50 MBW test occasions were systematically reviewed for technical elements and whether the breathing pattern was representative of relaxed tidal breathing by an experienced MBW operator. The impact of qualitative and quantitative criteria on inter-observer agreement was assessed across eight MBW operators (n = 20 test occasions, compared using a Kappa statistic).

**Results:**

Using qualitative criteria, 46/168 trials were rejected: 16.6% were technically unacceptable and 10.7% were excluded due to inappropriate breathing pattern. Reviewer agreement was good using qualitative criteria and further improved with quantitative criteria from (κ = 0.53–0.83%) to (κ 0.73–0.97%), but at the cost of exclusion of further test occasions in this retrospective data analysis.

**Conclusions:**

The application of the systematic review improved inter-observer agreement but did not affect reported MBW outcomes.

## Introduction

The lung clearance index (LCI), derived from the multiple breath inert gas washout (MBW) test, has been shown to be feasible across all ages and to detect early pathologic changes associated with obstructive lung disease.[1, 2] The availability of commercial MBW devices provides an opportunity to use the LCI as an outcome measure in multi-center randomized control trial, as well as for the routine assessment of patients in the clinical setting.[3, 4] While the MBW test requires minimal cooperation of the subject and is relatively easy to perform, accurate estimates of MBW outcomes rely on correct operation of the device, appropriate distraction of the subject to ensure they breathe in a manner representative of their relaxed tidal breathing pattern, and appropriate interpretation of the acquired data.[5]

The 2013 European Respiratory Society/American Thoracic Society (ERS/ATS) consensus statement for inert gas washout measurement provides general recommendations on test procedure and helps to standardize MBW devices and outcomes. The consensus statement outlines the criteria for a technically acceptable washout test and states that MBW outcomes are to be reported as the average of three technically acceptable washout curves [5]. However, there is little guidance on how to apply these criteria practically to MBW data prospectively at the time of test or as part of a retrospective review. In addition, while it is widely accepted that a subject should breathe in a stable pattern representative of tidal breathing [6, 7], the evaluation of breathing pattern is largely subjective and may introduce bias when interpreting outcomes. In this study we aimed to 1) develop a protocol, which systematically evaluates MBW measurements for technical elements and stability of the breathing pattern, 2) explore quantitative criteria as an additional way to make the protocol more objective, 3) compare inter-reviewer agreement of MBW trials, and 4) assess whether application of the protocol reduces the variability of reported outcomes.

## Methods

### Study Population

A random number generator was used to select 50 test occasions from a previously published cross-sectional study [8]; 25 healthy children and 25 children with CF between the ages of 6 and 18 years were selected. The original study was approved by the research ethics board (REB) at the Hospital for Sick Children (HSC), Toronto, Canada (REB# 1000019945). Informed written consent was obtained from the parents or guardians of healthy children and children with CF, and written assent was obtained from subjects who were able to understand the proposed research and what was expected of them as research participants.

### MBW Measurements

Participants performed multiple breath nitrogen washout (MBN_2_W) using the Exhalyzer D^®^ washout device (EcoMedics AG, Duernten, Switzerland), and associated Spiroware software (version 3.1.6) until the operator determined that three good trials were obtained or the subject was unable to continue testing.

### Systematic Evaluation of MBW Trials

The ATS/ERS consensus document [5] was used as a guide to develop a check list and detailed step-wise approach to facilitate the review of each MBW trial (see OLS). All MBW attempts within each test occasion were then evaluated using the check list by an experienced reviewer (RJ). The experienced reviewer (RJ) was involved in the data acquisition from a sub-set of the subjects; however data acquisition occurred more than 3 years before this secondary review and thus was unlikely to have affected the subsequent interpretation. Each trial was evaluated for technical elements. Trials were deemed technically unacceptable if 1) the tracer gas did not re-equilibrate between trials, 2) there was clear evidence of a leak or 3) the trial did not meet end of test criteria (see Table 1 for further details). The remaining technically acceptable trials were systematically reviewed for appropriate breathing pattern (Table 1, OLS). Technically acceptable trials that did not have a breathing pattern representative of relaxed tidal breathing were deemed unacceptable. The only clinical information available to reviewers was the subjects’ age, height and weight.

**Table 1:** Summary of technical and qualitative criteria used to evaluate MBW tracings.

Criteria for a Technically Acceptable Test		
	ATS/ERS Consensus Statement	Additional Criteria
No evidence of leak	A sudden spike in N_2_ concentration during inspirationPremature rise in N_2_ signal early in expirogram where N_2_ should be zero, or a decrease in airway dead space volumeA sudden step change in volume time trace or a step-up in N_2_ concentration plotted versus turn over (TO).	Deviations in the N_2_, O_2_ or CO_2_ signals inconsistent with the phase of the breath (inspiration/expiration).An increase in concentration of N_2_ due to dilution of oxygen without a corresponding increase in carbon dioxide.Loss of decay or prolonged plateau in end-tidal concentration of N_2_. Concentration of N_2_ does not return to zero on inspiration.
End of test criteria not met	Three consecutive breaths where the normalized end-tidal concentration of N_2_ fell below 2.5%	All three breaths should be tidal breaths appropriate for the subjects size*
Inadequate time between trials	Sufficient interval between runs when using resident inert gases to allow inert gas concentration to return to baseline values	End-tidal concentration of N_2_ at start of first trial of test session ≥77%Starting end-tidal concentration of N_2_ of subsequent trials within 1.5% of baseline
Evaluation of Breathing Pattern		
Pre-Phase: Tidal Volume (V_T_)& End expiratory lung volume (EELV)	Stable V_T_ and EELV over the preceding 30 s prior to start washout.No small volume breath immediately prior to start of washout Deviation in EELV at start of washout within 10% of mean V_T_ of 5 breaths immediately preceding the start of washout	Exhaled V_T_ for the last 5 breaths of the pre-phase appropriate for the subjects size*No sigh or very small breath immediately preceding washout rejected. No acute shift in volume/time trace over last 5 breaths of pre-phase.
Washout: Tidal Volume (V_T_)& End expiratory lung volume (EELV)	EELV is stable during washout Stable volume drift is acceptable; a sudden step change in volume time trace is acceptable provided leak was ruled out	V_T_ throughout the washout are appropriate for the subjects size*Minor deviations from V_T_ (i.e. swallow) are acceptable
Flow	No coughing	Stable respiratory flow rate with passive expiratory phase throughout the test.No evidence of forced exhalation; minor deviations (i.e. short breath hold) acceptable.
Trapped Gas Release	No evidence of significant trapped gas release with larger breaths.	If sigh or large breath, there is no evidence of increase in N_2_ signal over and above variability produced by tidal breaths.
Respiratory rate		No evidence of hyper or hypoventilation based on progression of end-tidal carbon dioxide concentration
Repeatability of FRC	FRC within 25% of the mean FRC of all technically acceptable trials	

*10–15ml/kg of 10^th^ -90^th^ % ideal body weight where ideal body weight was defined using the CDC pediatric growth charts [9]

### Quantitative Criteria

Data from both healthy children and children with CF were used to develop a quantitative quality control criterion. Variability of V_T_ was first quantified by calculating the within-trial coefficient of variation (CV) % of the exhaled V_T_ using tabular breath by breath data generated by the Spiroware software. Pre-phase breaths were not included in the calculation, nor were small breaths (tidal volume <3mL/kg). We then used the distribution of the CV V_T_ in acceptable and unacceptable trials to determine a cut-off which distinguished between acceptable and unacceptable data. Trials with values above the cut-off (≥ 20%) were deemed unacceptable. We determined ideal tidal volume (10–15ml/kg) for each subject assuming an ideal body weight for the subject’s age; ideal body weight was defined as 10^th^-90^th^ weight-for-age percentile using the CDC growth charts as a reference [9]. Thus the range of ideal tidal volume for each subject was calculated as 10ml * weight at the 10^th^ centile-for-age, and 15ml * weight at the 90^th^ centile-for-age. Finally, we calculated repeatability for FRC as recommended by the ATS/ERS consensus statement, whereas for LCI we used the first acceptable trial as a bench mark to calculate the relative difference in LCI for all subsequent trials.

### Statistical Analysis

#### Inter-Reviewer Agreement

To assess the agreement between reviewers a sub-set of 20 test occasions were reviewed by 8 MBW operators from 4 institutions (MP, RF, KR, CS, PR, MK, KH, and HW). Reviewers were asked to independently evaluate each tracing, identifying trials that were technically acceptable with an appropriate breathing pattern using the qualitative criteria described in Table 1 as well as the check list in S1 File. We compared inter-reviewer agreement for the decision to accept/reject between the primary reviewer (RJ) and each of the 8 reviewers using the Kappa (κ) statistic.

#### Comparison of Outcomes

To evaluate whether the systematic quality control review improved the precision of the LCI, we compared the within-test coefficient of variation (standard deviation/mean) and the inter-class correlation coefficient (ICC) of LCI from all trials to those classified as acceptable after qualitative review by the primary reviewer (RJ) and after the addition of the quantitative criteria. We also compared the average LCI calculated from all trials to the average LCI calculated after qualitative and quantitative review using a paired t-test.

Statistical analyses were done using Stata 13.0 (StataCorp 2013).

## Results

### Study Population

Demographic characteristics of the study population are described in Table 2. The CF group was slightly older and had elevated LCI compared to the healthy children. A total of 168 complete MBW trials from the 50 test occasions (range of 2–7 trials per test occasion) were reviewed.

**Table 2:** Demographics of cross-sectional study population, with results from each step of the quality control criteria applied.

	Total (n = 50)	Healthy (n = 25)	CF (n = 25)	Δ CF- HC*
Age: mean (range)	14.2 (7.7, 17.8)	13.4 (7.7, 17.2)	15.1 (9.5, 17.8)	**1.7 (0.2, 3.1)**
Male subjects: n (%)	20 (40%)	11 (44%)	9 (36%)	-8% (-35, 63%)
N trials (Median; Range acceptable trials)	168 (2; 1–5)	80 (2; 1–5)	88 (2; 1–4)	
Trials excluded for technical reasons: n (%)	28 (17%)	11 (13%)	17 (21%)	8% (-3, 20%)
Trials excluded for breathing pattern after qualitative review: n (%)	18 (11%)	16 (18%)	2 (3%)	-15% (-24, 7%)
Trials excluded for breathing pattern using CV V_T_ >20%: n (%)	25 (15%)	14 (16%)	11 (14%)	-2% (-13, 9%)
Trials excluded for breathing pattern using V_T_ appropriate for size; n (%)	6 (3.6%)	2 (2.5%)	4 (4.5%)	2% (-7.5%, 3.5)
Trials not meeting 25% FRC criteria: n (%)	0	0	0	n/a
LCI (all n = 50): mean (SD)	9.9 (4.2)	6.8 (0.4)	13.0 (3.8)	**6.2 (4.6, 7.7)**
LCI (qualitative review n = 34): mean (SD)	10.2 (4.4)	6.9 (0.4)	13.5 (4.0)	**6.6 (4.6, 8.6)**
LCI (excluding CV V_T_ >20%: n = 24): mean (SD)	10.1 (4.5)	6.8 (0.4)	13.4 (4.2)	**6.7 (4.1, 9.2)**
LCI (excluding V_T_ not appropriate for size: n = 33): mean (SD)	10.2 (4.5)	6.9 (0.4)	13.8 (4.0)	**6.9 (4.9, 8.9)**

CF: Cystic Fibrosis

LCI: Lung Clearance Index

V_T_: Tidal volume

CV: Coefficient of Variation

SD: standard deviation

* bold values indicate results are statistically significant (p<0.05).

### Systematic Evaluation of MBW Trials

Of the 168 trials, 28 (16.6%) were technically unacceptable and a further 18 (10.7%) trials were found not to have a breathing pattern representative of a relaxed tidal breathing. Exclusion of these 46 trials led to the exclusion of 16 (32%) test occasions; where a successful test occasion required at least two acceptable trials. None of the 168 trials were outside the FRC exclusion limits proposed by the ATS/ERS consensus document (>25% of the mean FRC value). LCI values were more repeatable when acceptable trials were compared; 60% of acceptable trials were within 5% of the first, whereas this was only true for 6% of unacceptable trials. Eighty-five percent of all acceptable trials were within 10% of the first, compared with 22% of unacceptable trials. Eighty-eight percent of acceptable LCI trials were within 1 LCI unit of the first acceptable trial. Using qualitative criteria alone the agreement between the primary reviewer and each of the eight external reviewers was good (n = 61 trials from a sub-set of 20 test occasions (Table 3)). The kappa statistic was >60%, indicating strong agreement for the majority of results.[10]

**Table 3:** Inter-Reviewer Agreement after independent review of 20 test-occasions (61 trials). Quantitative limits were applied after qualitative review, however each of the two quantitative limits (%CV V_T_ and V_T_ limits for size) were applied independently.

	Agreement after Qualitative Review		Agreement after %CV V_T_ limits applied		Agreement after appropriate V_T_ limits applied	
Reviewer	%	Kappa Statistic (95% Confidence Interval)	%	Kappa Statistic (95% Confidence Interval)	%	Kappa Statistic (95% Confidence Interval)
1	90%	0.75 (0.56, 0.94)	90%	0.80 (0.65, 0.95)	93%	0.87 (0.75, 0.99)
2	85%	0.59 (0.35, 0.83)	87%	0.73 (0.56, 0.91)	92%	0.84 (0.70, 0.97)
3	85%	0.53 (0.27, 0.79)	87%	0.73 (0.56, 0.91)	93%	0.87 (0.75, 0.99)
4	90%	0.75 (0.56, 0.94)	90%	0.80 (0.65, 0.95)	92%	0.84 (0.70, 0.97)
5	92%	0.77 (0.58, 0.96)	92%	0.83 (0.69, 0.97)	92%	0.83 (0.69, 0.97)
6	95%	0.74 (0.54, 0.93)	92%	0.84 (0.70, 0.97)	95%	0.90 (0.79, 1.00)
7	93%	0.81 (0.63, 0.99)	95%	0.90 (0.79, 1.00)	98%	0.97 (0.90, 1.00)
8	95%	0.83 (0.64, 1.00)	96%	0.93 (0.83, 1.00)	98%	0.97 (0.90, 1.00)

### Impact of additional Quantitative Criteria

**Fig 1:**
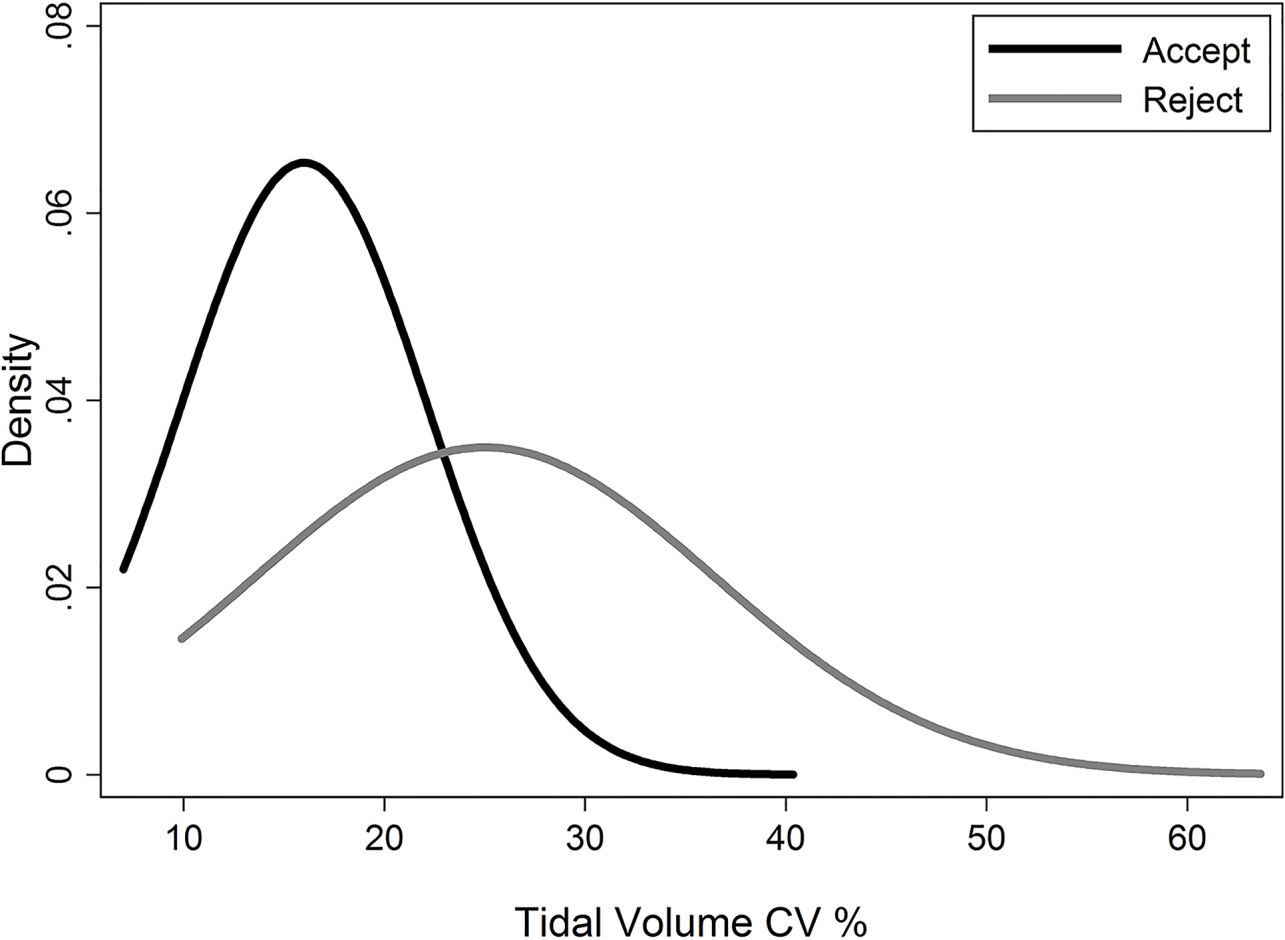
Distribution of CV% V_T_ for acceptable and unacceptable trials.

There was a significant difference in tidal volume variability (% CV V_T_) between acceptable and unacceptable trials (mean CV% 13.8 (SD 3.4) acceptable trials vs. 24.7 (SD 9.8) for unacceptable trials; non-parametric t-test p<0.001, Fig 1). The distribution of the V_T_ for acceptable trials (Range 7.0; 19.4) was used to define a cut-off of 20% as acceptable. When we applied the 20 CV% V_T_ cut-off to the dataset, an additional 25 trials and 10 test occasions were excluded. When we independently applied the limits of an appropriate V_T_ for subjects’ size to the dataset an additional 6 trials and 1 test occasion were excluded. Repeatability of LCI was similar when quantitative criteria were applied to define acceptable tests (data not shown). In all instances, regardless of training and experience, the addition of a quantitative component (either CV V_T_% or mean V_T_ appropriate for size), improved the agreement between reviewers (Table 3). The kappa statistic was >80%, indicating almost perfect agreement for the majority of results.[10]

### Comparison of Outcomes

Application of neither qualitative review nor quantitative criterion for breathing pattern stability changed the interpretation of group differences between health and disease: The difference in LCI between health and disease using all data was 6.2 (95% CI 4.6; 7.7, n = 50), compared with 6.6 (4.6; 8.6, n = 34) after qualitative review (Table 2). Similar results were observed when each of the two quantitative criterion for breathing pattern stability were applied (Table 2). The average LCI calculated as the mean of all trials (LCI = 9.9 (SD = 4.2), n = 50) was similar to the mean calculated from trials that were deemed acceptable after qualitative review (10.2 (SD = 4.4), n = 34) and when both quantitative criterion were applied (CV_VT_ 10.1 (4.5) and V_T_ size 10.2(4.5)). In a sub-set of subjects that had acceptable and unacceptable trials within the same test occasion, the exclusion of unacceptable changed the LCI between -0.2 units to 0.5 units. The within-subject within-test CV of the LCI for all trials was 5.1% (SD 4.4), and was smaller after qualitative review 3.3% (SD 2.5), and after we applied each of the two quantitative criterion (CV V_T_ 4.2% (SD 2.6%), V_T_ size 3.6% (SD 2.4)). There was no statistical difference between the LCI if two acceptable trials were reported instead of three [n = 31, mean difference 0.04 (-0.04; 0.11), p = 0.3490]. The systematic evaluation of MBW trials marginally improved the precision of the reported LCI values (ICC 98.6 vs. 95.0).

## Discussion

In this study we used the ATS/ERS MBW consensus statement as a basis to further develop a protocol to systematically evaluate MBW trials for technical criteria and appropriate breathing pattern. The systematic evaluation of MBW trials did not affect the reported LCI value on a group level, but it did change individual values. Overall there was good inter-review agreement between multiple reviewers after qualitative review, which was improved with the addition of a quantitative criterion for breathing stability. Systematic review of MBW trials could facilitate improved standardization between groups and consequently improve the reporting and interpretation of MBW outcomes.

In addition to correct operation of the device and technical considerations of the MBW test, accurate interpretation of results depends on the subject’s breathing pattern. The MBW test aims to evaluate the evenness of gas mixing within the lungs during normal tidal breathing at resting lung volume. Therefore it is essential that the subject breathes in their ‘normal’ manner during the test. However, it may be unreasonable to expect subjects to breathe in a perfectly stable and repeatable breathing pattern during washout and some degree of irregularity must be accepted. We sought to standardize the subjective review of breathing pattern, and developed a threshold for acceptability based on CV V_T_. Application of this additional criterion led to improved agreement between observers, albeit at the cost of feasibility. Conversely, excluding trials where the tidal volume was not appropriate for size improved inter-reviewer agreement and did not exclude many test occasions. Verbanck et al., used a fixed volume protocol to standardize tidal volume (and therefore breathing pattern) during MBW, whereby subjects were instructed to breathe at a tidal volume of 1L.[11] While this protocol may stabilize breathing, it is challenging in younger children, and the 1L target is not appropriate. Yammine et al., found that the fixed 1L protocol did minimize the % CV V_T_, but that restricting paediatric subjects to breathe at a fixed volume leads to statistical and clinical over-estimation of the LCI and changes in the FRC [12]. In an attempt to add objective assessment of breathing pattern, we chose a different approach, which focused on the correct identification of those trials with a stable breathing pattern. As this was a retrospective review of an existing dataset, where data were collected before the ATS/ERS consensus statement was published, it may not reflect what would happen in a prospective study. Quantitative limits for V_T_ size and variability built into commercial software may provide useful reminders to MBW operators and encourage them to collect additional during which the subject breathes within the suggested limits. In addition all of the reviewers were experienced testers, familiar with respiratory physiology. It has yet to be determined whether the same would apply to less experienced MBW operators.

The ATS/ERS consensus statement includes broad recommendations for a technically acceptable trial, including that any trial with an FRC greater than 25% different from the median should be excluded. In this study, there were no test occasions where the FRC was ≥ 25% of the median FRC. Reliance on the FRC criteria alone would have led to inclusion of a significant proportion (16.6%) of trials in which the breathing pattern was deemed not appropriate. LCI repeatability showed that acceptable MBW trials were more repeatable; however, just as is the case for spirometry, repeatability of an outcome should only be considered if the test was technically sound. In our analysis, we did not see changes in the average LCI, suggesting that LCI is a robust outcome for use in cross-sectional studies, whereas the differences observed within an individual subject suggest that impact of test quality will be larger within longitudinal studies or when treatment effects are measured using change in LCI.

The shape of the MBW curve is generated from a series of complex physiologic mechanisms and technical components. Furthermore, multiple breathing events can occur within a single MBW washout and each can influence the outcome independently; thus the impact on the LCI is unpredictable. Several research studies report quality control steps for MBW data, including identification of leaks, sighs and irregular breathing pattern; however specific details and quantitative limits are rarely included in the reports. The importance of signal alignment and software settings has also recently been demonstrated, highlighting the importance of correct operation of the equipment.[13] The overarching message from these available studies confirms that accurate interpretation of MBW outcomes requires a standardized protocol for the operation and interpretation of MBW tests. This is particularly relevant for longitudinal studies, where the variability of the outcome within and between subjects will affect interpretation of the results. The proposed qualitative review and quantitative criterion for breathing pattern stability need to be validated prospectively in a larger and longitudinal dataset.

### Limitations

We developed a protocol to systematically evaluate MBW tracings based on available evidence and tested it with knowledgeable MBW operators. Our findings are limited to a single dataset of 50 test occasions collected many years ago at a center and thus require further validation in other MBW outcomes, age groups, disease severities, disease groups, subject MBW testing experience, and testing in interventional studies. Our findings are not directly transferrable to other MBW set-ups (i.e. nitrogen washout by other manufactures or where SF_6_ is used as a tracer gas).

## Conclusions

As the MBW test moves from the research setting into clinical practice, there is a growing need to standardize measurement protocols, as well as interpretation strategies using a similar format to what exists for other pulmonary function tests, such as spirometry. This study provides a tool which can be used to further standardize reporting and interpretation of MBW outcomes.

## Supporting Information

S1 File Detailed description of qualitative quality control check list with accompanying worksheet. (PDF)
