# Supplementary material for: A Systematic Approach to Multiple Breath Nitrogen Washout Test Quality
Source: PLoS One. 2016 Jun 15;11(6):e0157523. doi: 10.1371/journal.pone.0157523 (PMC4909265; doi:10.1371/journal.pone.0157523)
Supplement: S1 File — (PDF) [file pone.0157523.s001.pdf]

Detailed Description of Qualitative Review

1. Pre-phase Breathing Pattern:
   a. Tidal volume (Vt)
      1. Is Vt appropriate for subject size (10-15 ml/kg ideal body weight)
      2. Is Vt stable (consistent volume breath to breath) for 5 breaths immediately prior to start of washout?
      3. Is the last breath of the pre-phase irregular (i.e. swallow, very small or very large)?
   b. End expiratory lung volume (EELV)
      1. Is EELV stable?
      2. Is volume time trace visually stable?
      3. Is deviation in EELV at start of washout within 10% of mean Vt of 5 breaths immediately preceding the start of washout?
   c. Time between trials
      1. Is starting end-tidal concentration of N_2 at baseline (first trial of test session) ≥77%
      2. Is starting end-tidal concentration of N_2 of subsequent trials within 1.5% of baseline?

2. Washout Breathing Pattern should be assessed for:
   a. Tidal Volume
      1. Is Vt appropriate for subject size (10-15 ml/kg ideal body weight)?
      2. Is the first breath of the washout irregular (i.e. swallow, very small or very large)?
      3. Is Vt stable over the washout?
      4. Are there any episodes of panting (< 0.5 x mean Vt) or a sighing (1.5 x mean Vt)?
         a. If there is an episode of panting has the software wrongly assumed end of test? (ie. 3 small breaths where $CetN_2$% < target)
         b. If there is a sigh is there any evidence of trapped gas release?
   b. EELV
      1. Is EELV (according to volume time trace) stable throughout the washout?
      2. Is there any evidence of breath stacking or incomplete exhalation in the volume trace?
   c. Flow
      1. Is the flow signal stable (PIF/PEF) with passive expiratory phase (no forced exhalation)?
      2. Does inspiratory flow ever go above 1000 ml/sec?
         If yes – is there a leak?

3. Based on the flow signal is there a stable respiratory rate across the washout?
4. Is there evidence of breath holding, swallow or other deviation from tidal breathing?
5. Is there evidence of a cough?

d. Hyper/hypoventilation
   1. Is the $CetCO_2$ between 4-6% over the washout?
   2. Is the CO_2 tracing stable?
      a. Is the $CetCO_2$ variable? SD of $etCO_2$ is running calc. of 3 breaths and will trigger error when SD > 0.25%
      b. Is the $CetCO_2$ continuously increasing or decreasing across the washout?

e. Leak
   1. On closer inspection, is there evidence of a leak?

Study ID: __ __ __ __ __ Visit: ____ Set:___ Filter: ___ Interface: ___ Barometric Pressure: ___________ Temperature: _________ Humidity: _________

Trial	Initial Assessment	Volume		Overall	Grade
**Pre-Washout**<br>______<br>Trial<br>______<br>Initials	Constant Decay [N2]? __________<br>Suspected Leak?	Vt appropriate for size?<br>Vt w/in 10-15 ml/kg target x 5 breaths?<br>Vt stable x 5 breaths?	Stable EELV for 5 breaths?	Initial $[N_2]$_______<br>Baseline =<br>FRC_________________________<br>Number of Breaths to washout ______	
	**Volume**		**Flow**		
**Washout**	Vt Stable?<br>Sigh?<br>If yes, at what point during the washout?	EELV stable?<br>Step Change EELV?<br>If Yes, breath stacking?	Stable?<br>Relaxed?<br>Respiratory Rate?<br>Breath Hold? Number of Times:<br>Cough?	[CO2] stable & between 4-6%?<br>Leak?<br>End of Test Criteria Met?	

Trial		Volume		Overall	Grade
**Pre-Washout**<br>______<br>Trial<br>______<br>Initials	Constant Decay [N2]? __________<br>Suspected Leak?	Vt appropriate for size?<br>Vt w/in 10-15 ml/kg target x 5 breaths?<br>Vt stable x 5 breaths?	Stable EELV for 5 breaths?	Initial $[N_2]$_____________<br>FRC_________________________<br>Number of Breaths to washout ______	
	**Volume**		**Flow**		
**Washout**	Vt Stable?<br>Sigh?<br>If yes, at what point during the washout?	EELV stable?<br>Step Change EELV?<br>If Yes, breath stacking?	Stable?<br>Relaxed?<br>Respiratory Rate?<br>Breath Hold? Number of Times:<br>Cough?	[CO2] stable & between 4-6%?<br>Leak?<br>End of Test Criteria Met?	
